# An Entropy Formulation Based on the Generalized Liouville Fractional Derivative

**DOI:** 10.3390/e21070638

**Published:** 2019-06-28

**Authors:** Rui A. C. Ferreira, J. Tenreiro Machado

**Affiliations:** 1 Grupo Física-Matemática, Faculdade de Ciências, Universidade de Lisboa, Avenida Professor Gama Pinto, 2, 1649-003 Lisboa, Portugal; 2 Institute of Engineering, Polytechnic of Porto, Department of Electrical Engineering, R. Dr. António Bernardino de Almeida, 431, 4249-015 Porto, Portugal

**Keywords:** fractional derivatives, fractional calculus, entropy

## Abstract

This paper presents a new formula for the entropy of a distribution, that is conceived having in mind the Liouville fractional derivative. For illustrating the new concept, the proposed definition is applied to the Dow Jones Industrial Average. Moreover, the Jensen-Shannon divergence is also generalized and its variation with the fractional order is tested for the time series.

## 1. Introduction

The quest of generalizing the Boltzmann–Gibbs entropy has become an active field of research in the past 30 years. Indeed, many formulations appeared in the literature extending the well-known formula (see e.g., [1,2,3,4]):

(1) $$S\left(p\right)=\sum_{i}-\ln\left(p_{i}\right)p_{i}.$$

The (theoretical) approaches to generalize Equation (1) may vary considerably (see e.g., [1,2,3]). In this work we are particularly interested in the method firstly proposed by Abe in [1], which consists of the basic idea of rewriting Equation (1) as

(2) $$S\left(p\right)={\left.\sum_{i}-\frac{d}{dt}p_{i}^{t}\right|}_{t=1}={\left.\sum_{i}\frac{d}{dt}p_{i}^{-t}\right|}_{t=-1}.$$

Concretely, we substitute the differential operator $\frac{d}{dt}$ in Equation (2) by a suitable *fractional* one (see Section 2 for the details) and then, after some calculations, we obtain a novel (at least to the best of our knowledge) formula, which depends on a parameter 0<α≤1.

The paper is structured as follows. Section 2 introduces and discusses the motivation for the new entropy formulation. Section 3 analyses the Dow Jones Industrial Average. Additionally, The Jensen-Shannon divergence is also adopted in conjunction with the hierarchical clustering technique for analyzing regularities embedded in the time series. Finally, Section 4 outlines the conclusions.

## 2. Motivation

Let us introduce the following entropy function:

(3) $$S_{\alpha }\left(p\right)=\sum_{i}\frac{\Gamma (1-\ln\left(p_{i}\right))}{\Gamma (1-\ln\left(p_{i}\right)-\alpha )}p_{i},\;\alpha \in \left(0,1\right],$$

where $\Gamma \left(t\right)=\int_{0}^{\infty }x^{t-1}e^{-x}dx$ is the gamma function.

**Figure 1:**
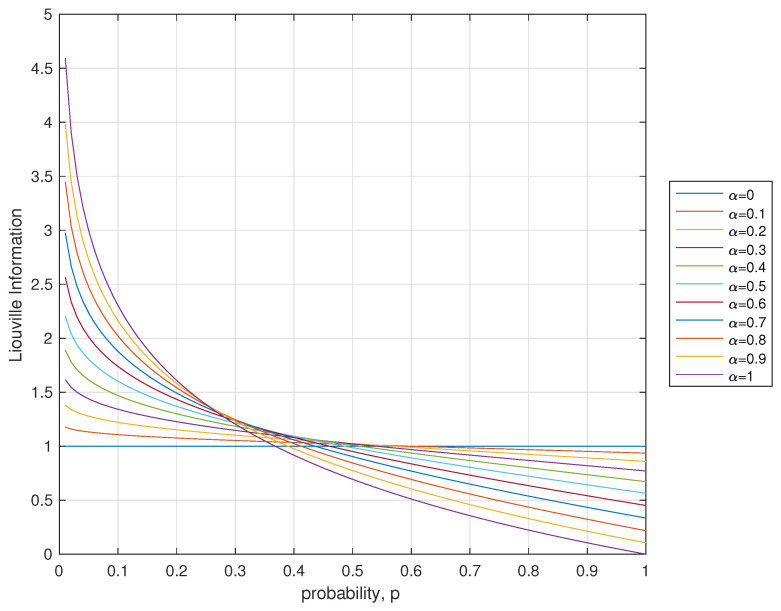
Liouville information $I_{\alpha }\left(p_{i}\right)=\frac{\Gamma (1-\ln\left(p_{i}\right))}{\Gamma (1-\ln\left(p_{i}\right)-\alpha )}$.

We define the quantity $I_{\alpha }\left(p_{i}\right)=\frac{\Gamma (1-\ln\left(p_{i}\right))}{\Gamma (1-\ln\left(p_{i}\right)-\alpha )}$ as the Liouville information (See Figure 1).

Our motivation to define the entropy function given by Equation (3) is essentially due to the works of Abe [1] and Ubriaco [4]. Indeed, in Section 3 of [4], the author notes (based on Abe’s work [1]) that the Boltzmann-Gibbs and the Tsallis entropies may be obtained by

$$S={\left.\sum_{i}\frac{d}{dt}p_{i}^{-t}\right|}_{t=-1},$$

and

$$S={\left.\sum_{i}\frac{d}{d_{q}t}p_{i}^{-t}\right|}_{t=-1},\;\mathrm{where}\frac{d}{d_{q}t}f=\frac{f(qt)-f(t)}{(q-1)t},$$

respectively. From this, he substitutes the above differential operator by a Liouville fractional derivative (see Section 2.3 in [5]) and then he defines a *fractional* entropy (see (19) in [4]). With this in mind and taking into account the generalization of the Liouville fractional derivative given by the “fractional derivative of a function with respect to another function” (see Section 2.5 in [5]) we consider using it in order to define a novel entropy. The Liouville fractional derivative of a function *f* with respect to another function *g* (with $g^{\prime }>0$) is defined by [5,6],

(4) $$D_{g}^{\alpha }f\left(t\right)=\frac{1}{\Gamma \left(1-\alpha \right)g^{\prime }\left(t\right)}\frac{d}{dt}\int_{-\infty }^{t}{\left[g\left(t\right)-g\left(s\right)\right]}^{-\alpha }g^{\prime }\left(s\right)f\left(s\right)ds,\;0<\alpha \leq 1.$$

It is important to keep in mind that our goal is to obtain an explicit formula for the entropy. Therefore, we can think that a “good” candidate for *g* is the exponential function, due to the fact that $p_{i}^{-t}=e^{-t\ln(p_{i})}$ and also the structure of Equation (4). We chose $g\left(x\right)=e^{x+1}$. Let us then calculate Equation (4) with $f\left(t\right)=p_{i}^{-t}$ and $g\left(x\right)=e^{x+1}$. We obtain

$$\begin{array}{cc} D_{g}^{\alpha }f\left(t\right) & =\frac{1}{\Gamma \left(1-\alpha \right)e^{t+1}}\frac{d}{dt}\int_{-\infty }^{t}{\left[e^{t+1}-e^{s+1}\right]}^{-\alpha }e^{s+1}e^{-s\ln(p_{i})}ds\\ & =\frac{1}{\Gamma \left(1-\alpha \right)e^{t+1}}\frac{d}{dt}e^{-\alpha (t+1)}\int_{-\infty }^{t}{\left[1-e^{s-t}\right]}^{-\alpha }e^{s(1-\ln\left(p_{i}\right))+1}ds\\ & =\frac{1}{\Gamma \left(1-\alpha \right)e^{t+1}}\frac{d}{dt}e^{-\alpha (t+1)}\int_{0}^{1}{\left(1-u\right)}^{-\alpha }e^{\left(t+\ln\left(u\right)\right)\left(1-\ln\left(p_{i}\right)\right)+1}\frac{du}{u}\\ & =\frac{1}{\Gamma \left(1-\alpha \right)e^{t+1}}\frac{d}{dt}e^{-\alpha \left(t+1\right)+t(1-\ln\left(p_{i}\right))+1}\int_{0}^{1}{\left(1-u\right)}^{-\alpha }u^{-\ln(p_{i})}du\\ & =\frac{-\alpha +1-\ln(p_{i})}{\Gamma \left(1-\alpha \right)e^{t+1}}e^{-\alpha \left(t+1\right)+t(1-\ln\left(p_{i}\right))+1}\frac{\Gamma \left(1-\alpha \right)\Gamma (1-\ln\left(p_{i}\right))}{\Gamma (2-\alpha -\ln\left(p_{i}\right))}\\ & =\left(1-\alpha -\ln\left(p_{i}\right)\right)e^{\left(-\alpha -1\right)\left(t+1\right)+t(1-\ln\left(p_{i}\right))+1}\frac{\Gamma (1-\ln\left(p_{i}\right))}{\Gamma (2-\alpha -\ln\left(p_{i}\right))}. \end{array}$$

It follows that,

$$D_{g}^{\alpha }f\left(-1\right)=\left(1-\alpha -\ln\left(p_{i}\right)\right)p_{i}\frac{\Gamma (1-\ln\left(p_{i}\right))}{\Gamma (2-\alpha -\ln\left(p_{i}\right))},$$

and after using the property Γ(x+1)=xΓ(x),x>0, we finally get

$$D_{g}^{\alpha }f\left(-1\right)=p_{i}\frac{\Gamma (1-\ln\left(p_{i}\right))}{\Gamma (1-\alpha -\ln\left(p_{i}\right))}.$$

We have, therefore, motivated the definition provided in Equation (3).

**Remark** **1.** *We note that, if $p_{i}=1$ for some i∈N and α=1, then in Equation (3) we have a division by zero. In this case we are obviously thinking about the limit of that function, i.e.,*

$$\lim_{\left(x,\alpha )\to (1,1\right)}\frac{\Gamma (1-\ln(x))}{\Gamma (1-\ln(x)-\alpha )}x=0.$$

*In addition, it is not hard to check that, for 0<α≤1, we have*

$$\lim_{x\to 0^{+}}\frac{\Gamma (1-\ln(x))}{\Gamma (1-\ln(x)-\alpha )}x=0.$$

*Therefore, we put in Equation* (3): *$\frac{\Gamma (1-\ln(0))}{\Gamma (1-\ln(0)-\alpha )}0=0$, with 0<α≤1.*

**Remark** **2.** *The entropy function defined in Equation* (3) *brings interesting challenges. For instance, though numerically the function*

$$\frac{\Gamma (1-\ln(x))}{\Gamma (1-\ln(x)-\alpha )}x,\;x\in \left(0,1\right)$$

**Figure 2:**
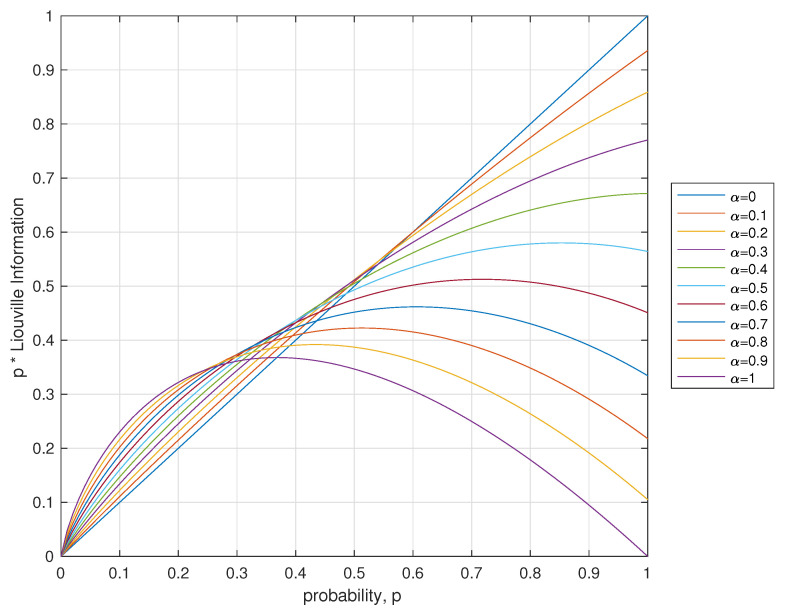
Plot of $p_{i}\frac{\Gamma (1-\ln\left(p_{i}\right))}{\Gamma (1-\ln\left(p_{i}\right)-\alpha )}$ versus $p_{i}$.

*for α∈(0,1) seems to be concave (see Figure 2), a rigorous proof of that fact was not yet obtained.*

## 3. An Example of Application

The Dow Jones Industrial Average (DJIA) is an index based on the value of 30 large companies from the United States traded in the stock market during time. The DJIA and other financial indices reveal a fractal nature and has been the topic of many studies using distinct mathematical and computational tools [7,8]. In this section we apply the previous concepts in the study of the DJIA in order to verify the variation of the new expressions with the fractional order. Therefore, we start by analyzing the evolution of daily closing values of the DJIA from January 1, 1985, to April 5, 2019, in the perspective of Equation (3). All weeks include five days and missing values corresponding to special days are interpolated between adjacent values. For calculating the entropy we consider time windows of 149 days performing a total of n=34 years.

**Figure 3:**
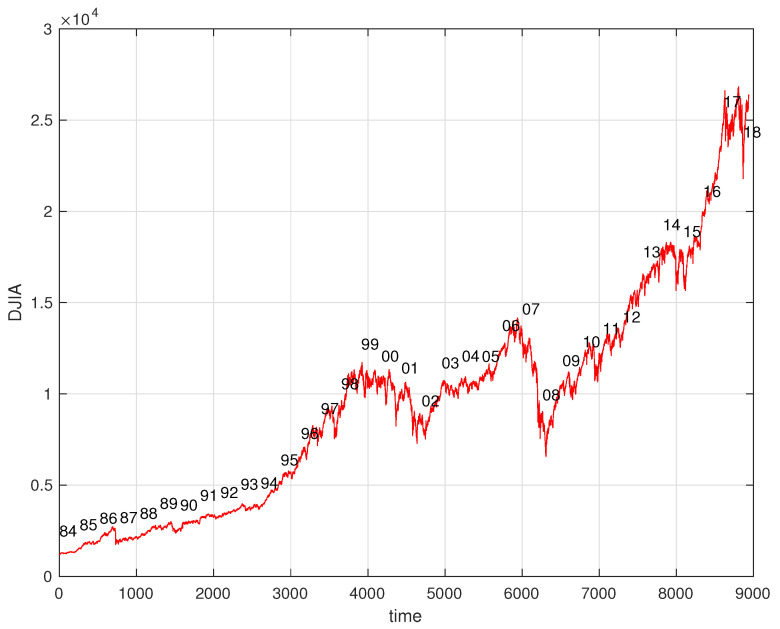
Evolution of the Dow Jones Industrial Average (DJIA) versus time *t* from January 1, 1985, to April 5, 2019.

**Figure 4:**
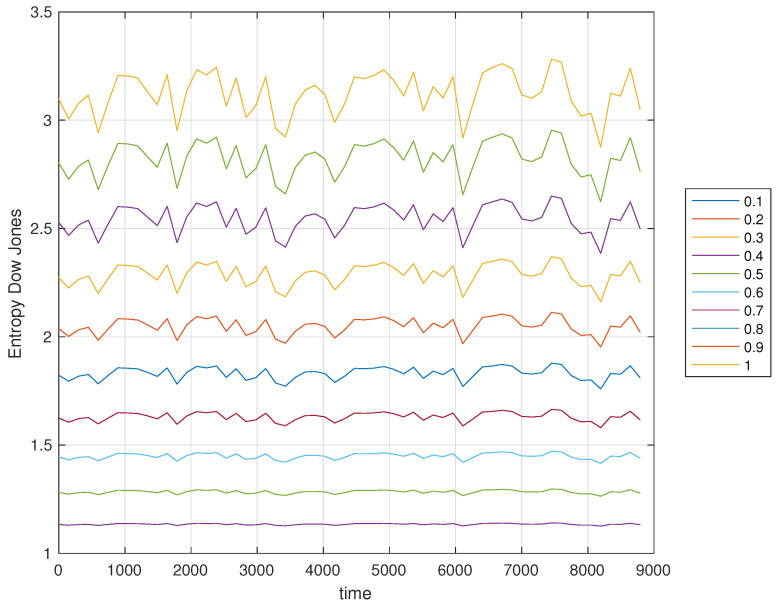
Evolution of the $S_{\alpha }\left(t\right)$ versus time *t* from January 1, 1985, to April 5, 2019, and $\alpha =\left\{0,0.1,\ldots ,0.9,1\right\}$.

Figure 3 and Figure 4 show the time evolution of the DJIA and the corresponding value of $S_{\alpha }$ for $\alpha =\left\{0,0.1,\ldots ,0.9,1\right\}$.

We verify that $S_{\alpha }\left(t\right)$ has a smooth evolution with α that plays the role of a parameters for adjusting the sensitivity of the entropy index.

The Jensen-Shannon divergence (JSD) measures the similarity between two probability distributions and is given by

(5) $$JSD\left(P||Q\right)=\frac{1}{2}D\left(P||M\right)+\frac{1}{2}D\left(Q||M\right),$$

where $M=\frac{1}{2}\left(P+Q\right)$, and $D\left(P||M\right)$ and $D\left(Q||M\right)$ represent the Kullback-Leibler divergence between distributions *P* and *M*, and *P* and *Q*, respectively.

For the classical Shannon information $I\left(p_{i}\right)=-\log\left(p_{i}\right)$ the JSD can be calculated as:

(6) $$JSD\left(P||Q\right)=\frac{1}{2}\left(\sum_{i}p_{i}\log\left(p_{i}\right)+\sum_{i}q_{i}\log\left(q_{i}\right)\right)-\sum_{i}m_{i}\log\left(m_{i}\right).$$

In the case of the Liouville information $I_{\alpha }\left(p_{i}\right)=\frac{\Gamma (1-\ln\left(p_{i}\right))}{\Gamma (1-\ln\left(p_{i}\right)-\alpha )}$ the JSD can be calculated as:

(7) $$JSD\left(P||Q;\alpha \right)=\frac{1}{2}\left(\sum_{i}p_{i}\frac{\Gamma \left(1-\ln\left(p_{i}\right)\right)}{\Gamma \left(1-\ln\left(p_{i}\right)-\alpha \right)}+\sum_{i}q_{i}\frac{\Gamma \left(1-\ln\left(q_{i}\right)\right)}{\Gamma \left(1-\ln\left(q_{i}\right)-\alpha \right)}\right)-\sum_{i}m_{i}\frac{\Gamma \left(1-\ln\left(m_{i}\right)\right)}{\Gamma \left(1-\ln\left(m_{i}\right)-\alpha \right)}.$$

Obviously, for α=1 we obtain the Shannon formulation.

For processing the data produced by the JSD we adopt hierarchical clustering (HC). The main objective of the HC is to group together (or to place far apart) objects that are similar (or different) [9,10,11,12]. The HC receives as input a symmetrical matrix *D* of distances (e.g., the JSD) between the *n* items under analysis and produces as output a graph, in the form of a dendogram or a tree, where the length of the links represents the distance between data objects. We have two alternative algorithms, namely the agglomerative and the divisive clustering. In the first, each object starts in its own singleton cluster and, at each iteration of the HC scheme, the two most similar (in some sense) clusters are merged. The iterations stop when there is a single cluster containing all objects. In the second, all objects start in a single cluster and at each step, the HC removes the ‘outsiders’ from the least cohesive cluster. The iterations stop when each object is in its own singleton cluster. Both iterative schemes are achieved using an appropriate metric (a measure of the distance between pairs of objects) and a linkage criterion, which defines the dissimilarity between clusters as a function of the pairwise distance between objects.

In our case the objects correspond to the n=34 years, from January 1, 1985, to December 31, 2018, that are compared using the JSD and resulting matrix *D* (with dimension n×n) processed by means of HC.

**Figure 5:**
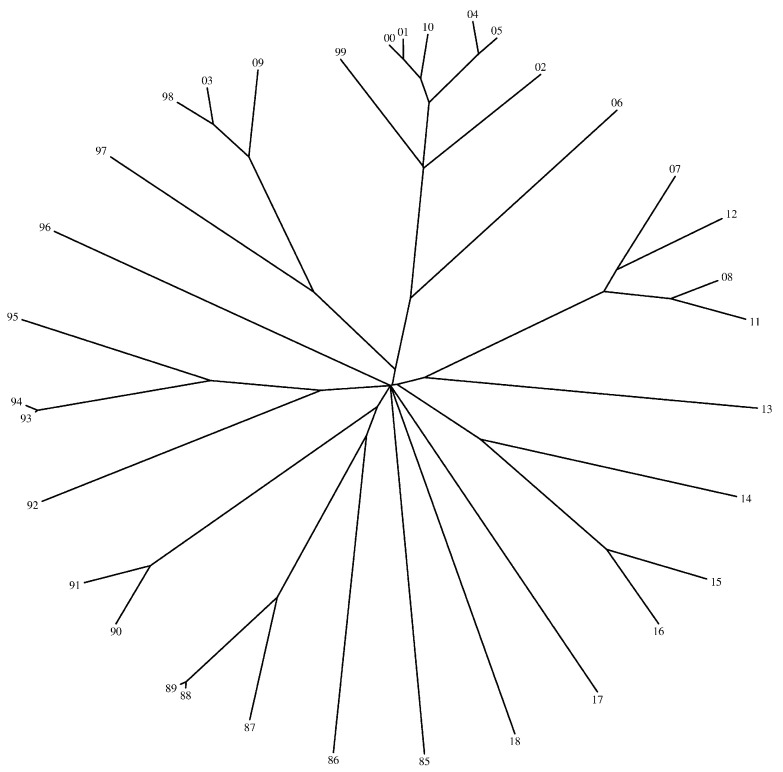
Tree generated by the hierarchical clustering for the Shannon JSD.

**Figure 6:**
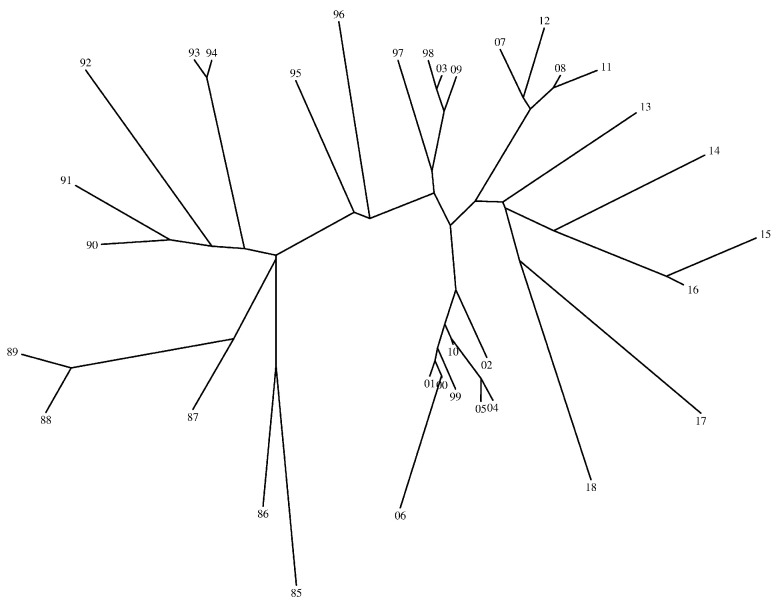
Tree generated by the hierarchical clustering for the Liouville JSD and α=0.1.

**Figure 7:**
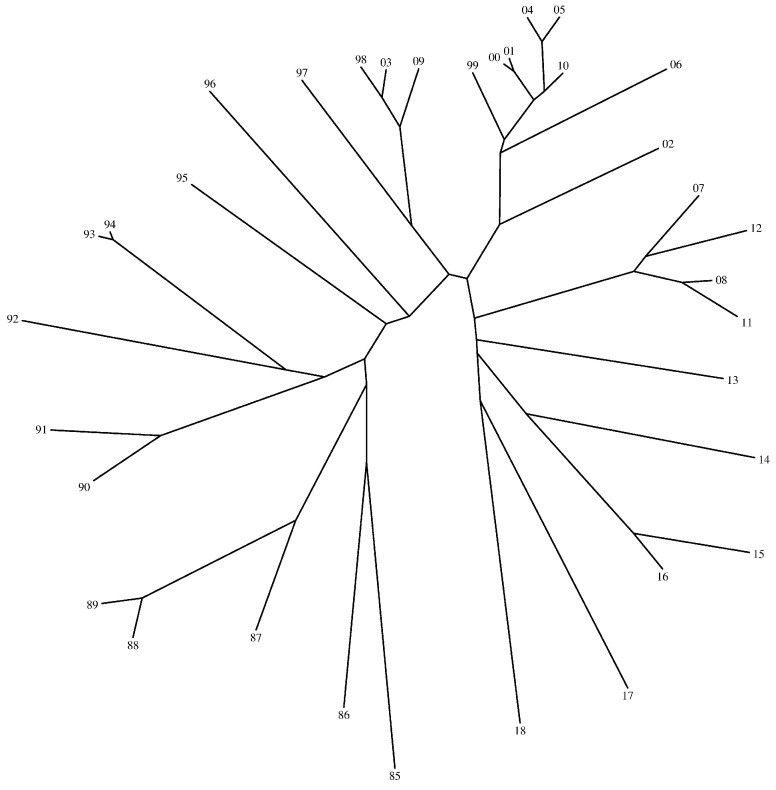
Tree generated by the hierarchical clustering for the Liouville JSD and α=0.5.

Figure 5, Figure 6 and Figure 7 show the trees generated by the hierarchical clustering for the Shannon and the Liouville Jensen-Shannon divergence measures (with $\alpha =\left\{0.1,0.5\right\}$), respectively. The 2-digit labels of the ‘leafs’ of the trees denote the years.

We note that our goal is not to characterize the dynamics of the DJIA time evolution since it is outside the scope of this paper. In fact, we adopt the DJIA simply as a prototype data series for assessing the effect of changing the value of α in the JSD and consequently in the HC generated tree. We verify that in general there is a strong similarity of the DJIA between consecutive years. In what concerns the use of the Shannon versus the Liouville JSD, we observe that for α close to 1 both entropies lead to identical results, while for α close to 0 the Liouville JSD produces a distinct tree. Therefore, we conclude that we can adjust the clustering performance by a proper tuning of the parameter α.

## 4. Conclusions

This paper presented a new formulation for entropy based on the (generalized) Liouville definition of fractional derivative. The generalization leads not only to a new entropy index, but also to novel expressions for fractional information and Jensen-Shannon divergence. The sensitivity of the proposed expression to variations of the fractional order is tested for the DJIA time series.
